# 76例初诊*EGFR*突变阳性合并胸腔积液肺腺癌患者的临床特征及预后分析一项单中心、回顾性研究

**DOI:** 10.3779/j.issn.1009-3419.2022.101.13

**Published:** 2022-03-20

**Authors:** 文琤 尹, 华 张, 阳春 顾, 福梅 易, 倩 李, 燕娥 刘, 艳红 姚, 镇涛 刘, 宝山 曹

**Affiliations:** 1 100191 北京，北京大学第三医院肿瘤化疗与放射病科 Department of Medical Oncology and Radiation Sickness, Peking University Third Hospital, Beijing 100191, China; 2 100191 北京，北京大学第三医院临床流行病学研究中心 Research Center of Clinical Epidemiology, Peking University Third Hospital, Beijing 100191, China

**Keywords:** 肺肿瘤, 表皮生长因子受体, 胸腔积液, 预后, Lung neoplasms, Epidermal growth factor receptor, Pleural effusion, Prognosis

## Abstract

**背景与目的:**

恶性胸腔积液是肺腺癌患者常见临床表现之一，肺腺癌初诊时合并胸腔积液提示预后不佳。表皮生长因子受体(epidermal growth factor receptor, *EGFR*)突变主要发生在肺腺癌患者中，不同亚型预后不同。初诊时不同亚型*EGFR*突变阳性合并胸腔积液肺腺癌患者的临床特征及预后因素目前尚不明确，本研究拟探讨此类患者的临床特征及预后影响因素，旨在为此类患者提供诊治参考。

**方法:**

回顾性分析2012年1月-2021年6月期间，北京大学第三医院肿瘤化疗与放射病科收治的初诊时*EGFR*突变阳性且合并胸腔积液肺腺癌患者的临床特征、治疗方法、治疗疗效和一线治疗中位无进展生存期(progression-free survival, PFS)，采用*Pearson*卡方检验或*Fisher*精确概率法进行组间比较，采用*Kaplan-Meier*法进行生存分析，采用*Cox*比例风险回归模型进行多因素分析。

**结果:**

共筛选出符合入组条件的患者76例，*EGFR*经典突变19del、21L858R和非经典突变的发生率分别为46.0%、38.2%和15.8%，3种突变亚型在性别、年龄、发病时有无呼吸困难、是否合并其他远处转移、胸腔积液部位、胸腔积液量、有无合并其他部位积液、肿瘤原发灶-淋巴结-转移(tumor-node-metastasis, TNM)分期、有无合并其他基因突变、胸腔积液的治疗方法等方面无显著差异(*P* > 0.05)。在*EGFR*经典突变19del、21L858R和非经典突变患者中，一线应用化疗的占比分别为17.1%、20.7%和58.3%(*P*=0.001)；一线疾病控制率分别为94.3%、75.9%和50.0%(*P*=0.003)；胸腔积液控制率分别为94.3%、79.3%和66.7%(*P*=0.04)；PFS分别为287 d、327 d和55 d(*P*=0.001)。单因素分析显示*EGFR*突变亚型、胸腔积液控制情况、一线治疗药物、一线治疗疗效与PFS显著相关(*P* < 0.05)，*Cox*多因素分析显示仅*EGFR*突变类型、一线治疗疗效是PFS的独立预后因素(*P* < 0.05)。

**结论:**

初诊*EGFR*突变阳性且合并胸腔积液的肺腺癌患者中，*EGFR*经典突变(19del和21L858R)患者的PFS显著优于非经典突变者，提升一线治疗疗效是改善此类患者预后的关键。

## Abstract (en)

**Background and objective:**

Malignant pleural effusion is one of the common clinical manifestations of patients with lung adenocarcinoma. Patients with pleural effusion at the initial diagnosis of lung adenocarcinoma usually indicate poor prognosis. Epidermal growth factor receptor (*EGFR*) mutations mainly occur in patients with lung adenocarcinoma. Patients with different mutant subtypes have different prognosis. The clinical characteristics and prognostic factors of patients with *EGFR* mutated lung adenocarcinoma of different molecular subtypes combined with pleural effusion at initial diagnosis are still unclear. This study was designed to explore the clinical characteristics and prognostic factors of these patients in order to provide management recommendations for them.

**Methods:**

A retrospective analysis of the clinical characteristics, treatment, outcomes and progression-free survival (PFS) of first-line treatment in patients with *EGFR* mutated lung adenocarcinoma combined with pleural effusion at initial diagnosis admitted to Department of Medical Oncology and Radiation Sickness, Peking University Third Hospital from January 2012 to June 2021 was performed. *Pearson's* chi-square test or *Fisher's* exact test were performed for comparison between groups. *Kaplan-Meier* method was performed for survival analysis and *Cox* proportional risk regression model was performed for multivariate analysis.

**Results:**

76 patients met the inclusion criteria in this study. The incidences of *EGFR* classical mutations 19del, 21L858R and non-classical mutations were 46.0%, 38.2% and 15.8%, respectively among these patients. There was no significant difference between the three mutations in terms of gender, age, presence of dyspnea at presentation, whether other distant metastases were combined, site of pleural effusion, volume of pleural effusion, presence of other combined effusions, tumor-node-metastasis (TNM) stage, presence of other gene mutations, and treatment of pleural effusion (*P* > 0.05). In patients with *EGFR* classical mutations 19del or 21L858R or non-classical mutations subtype, the proportion of chemotherapy in first-line regimens were 17.1%, 20.7% and 58.3%, respectively (*P*=0.001); and first-line disease control rates were 94.3%, 75.9% and 50%, respectively (*P*=0.003); pleural effusion control rates were 94.3%, 79.3% and 66.7%, respectively (*P*=0.04); PFS were 287 d, 327 d and 55 d, respectively (*P*=0.001). Univariate analysis showed that *EGFR* mutation subtype, control of pleural effusion, first-line treatment agents, and first-line treatment efficacy were significantly associated with PFS (*P* < 0.05). *Cox* multifactorial analysis showed that only *EGFR* mutation subtype and first-line treatment efficacy were independent prognostic factors for PFS (*P* < 0.05).

**Conclusion:**

PFS was significantly better for classical mutations than for non-classical mutations in patients with *EGFR* mutated lung adenocarcinoma combined with pleural effusion at initial diagnosis. Improving the efficacy of first-line therapy is the key to improve the prognosis of these patients.

肺癌是我国发病率、死亡率最高的恶性肿瘤^[1]^，15%的患者初诊时合并恶性胸腔积液^[2]^，大约20%的肺癌患者在首次接受计算机断层扫描（computed tomography, CT）检查时存在胸腔积液表现^[3]^。肺癌初诊合并恶性胸腔积液意味着预后不良，5年生存率不到3%^[4, 5]^。表皮生长因子受体（epidermal growth factor receptor, *EGFR*）的发现以及*EGFR*酪氨酸激酶抑制剂（tyrosine kinase inhibitors, TKIs）在*EGFR*突变阳性肺癌患者中疗效显著，开启了肺癌靶向治疗时代，EGFR-TKIs已成为晚期含有*EGFR*敏感突变肺癌患者的一线治疗首选。*EGFR*突变主要发生在肺腺癌患者中，恶性胸腔积液是肺腺癌患者常见的临床表现之一，*EGFR*突变肺腺癌或肺癌合并恶性胸腔积液的研究报道较多，但关于初诊*EGFR*突变阳性且合并恶性胸腔积液的肺腺癌患者预后的相关报道较少。

随着基因检测技术的发展，*EGFR*基因突变目前已知存在多种亚型，其中*EGFR*外显子19缺失突变（19del）和外显子21 L858R突变（21L858R）被称为*EGFR*经典突变，对EGFR-TKIs疗效反应较好；其他*EGFR*突变类型突变率低，又称为罕见突变或非经典突变，对EGFR-TKIs反应不一。关于初诊时不同*EGFR*突变亚型且合并胸腔积液的肺腺癌患者的临床特征、治疗情况以及预后因素等尚缺乏报道。本研究回顾性分析了北京大学第三医院肿瘤化疗与放射病科76例初诊时含有*EGFR*不同突变亚型且合并胸腔积液的肺腺癌患者的临床特征、治疗及预后因素，旨在为此类患者提供诊治参考。

## 1 资料与方法

### 1.1 研究对象

收集2012年1月-2021年6月期间北京大学第三医院肿瘤化疗与放射病科收治的肺癌患者，通过电子病历信息系统查询诊断中含有“胸腔积液”，同时满足以下纳入标准：①确诊时胸部CT检查示合并胸腔积液；②组织学或细胞学确诊为肺腺癌；③组织或细胞学基因检测证实存在*EGFR*突变；④至少接受过一线治疗；⑤具有较完整的初始分期及疗效评价时的影像学资料（如：胸腹部CT、脑核磁、骨扫描等）。排除标准：①非初诊时发生的恶性胸腔积液患者；②胸腔积液经证明为非恶性的患者；③无*EGFR*突变；④未接受过一线治疗；⑤临床资料缺乏者。

### 1.2 资料收集

采集并记录患者的临床资料，包括年龄、性别、发病时临床症状、肿瘤原发灶-淋巴结-转移（tumor-node-metastasis, TNM）分期、基因检测方法、基因检测结果、胸腔积液的影像学表现、胸腔积液的治疗方法、一线治疗方案、一线治疗疗效和一线治疗的无进展生存期（progression-free survival, PFS）。

### 1.3 评估标准

肿瘤分期依据国际肺癌研究协会颁布的第8版分期标准^[5]^。疗效评价依照实体瘤疗效评价标准（Response Evaluation Criteria in Solid Tumors, RECIST）1.1版本，分为完全缓解（complete response, CR）、部分缓解（partial response, PR）、疾病稳定（stable disease, SD）和疾病进展（progressive disease, PD）^[6]^。胸腔积液在诊断时均行16或64排螺旋CT明确，本研究中按胸腔积液在CT横断面上的最大深度，自定义为少量胸腔积液（最大深度≤2 cm）、中量胸腔积液（最大深度 > 2 cm， < 1/2胸腔）、大量胸腔积液（最大深度≥1/2胸腔）。一线治疗后胸腔积液深度增加20%定义为胸腔积液增加，减少20%定义为胸腔积液减少，增加或减少均不到20%定义为胸腔积液稳定，CT未显示胸腔积液定义为胸腔积液消失。

### 1.4 研究终点

主要研究终点是一线治疗的PFS，定义为从接受治疗开始至治疗后疾病进展或者因各种原因出现死亡的这段时间。次要研究终点是一线治疗的疾病控制率（disease control rate, DCR），DCR=（CR+PR+SD）例数/总例数。胸腔积液控制率定义为胸腔积液稳定、减少及消失且维持30 d以上的患者占全部患者的比例。

### 1.5 随访

通过病史查阅和电话随访两种方式收集资料。病史查阅是通过查阅患者住院及门急诊病历信息了解患者治疗及复发转移情况；电话随访是由专职人员根据住院信息记录的联系方式定期联系患者本人或家属了解其疗效及生存情况；随访截至2022年1月1日。

### 1.6 统计学方法

采用SPSS 24.0统计软件对数据进行统计学分析。对于偏态分布或分布情况不明的连续变量以中位数表示。用*Pearson*卡方检验或*Fisher*精确概率法进行组间比较。采用*Kaplan-Meier*法进行单因素生存分析，使用R语言（v4.3.4）绘制生存曲线，应用*Log-rank*法进行检验。采用*Cox*比例风险回归模型进行多因素分析。以*P* < 0.05为差异有统计学意义。

## 2 结果

### 2.1 76例初诊*EGFR*阳性合并胸腔积液肺腺癌患者的临床特征

共筛选肺癌合并胸腔积液患者297例，剔除小细胞肺癌45例，肺鳞癌60例，肺腺鳞癌2例，肺低分化癌3例，治疗进展后发生胸腔积液者35例，未进行一线治疗者6例，胸腔积液细胞学证实为非恶性者2例，无*EGFR*基因突变者68例，符合入组条件的患者共76例。76例患者中*EGFR*经典突变患者64例（84.2%），19del和21L858R突变分别为35例（46.0%）和29例（38.2%），非经典突变12例（15.8%）。具体临床特征见表 1。

**表 1:** 76例初诊*EGFR*阳性合并胸腔积液肺腺癌患者的临床特征[*n* (%)] Clinical characteristics of 76 lung adenocarcinoma patients harboring *EGFR* mutations with pleural effusion at initial diagnosis [*n* (%)]

Characteristics	Total (*n*=76)	*EGFR*			*P*
		19del (*n*=35)	21L858R (*n*=29)	Non-classical mutations (*n*=12)	
Gender					0.188
Male	32 (42.1)	14 (40.0)	10 (34.5)	8 (66.7)	
Female	44 (57.9)	21 (60.0)	19 (65.5)	4 (33.3)	
Age [median (range), yr]	65 (39-88)	65 (46-88)	65 (40-88)	65 (39-74)	0.502
≤65	39 (51.3)	16 (45.7)	15 (51.7)	8 (66.7)	
> 65	37 (48.7)	19 (54.3)	14 (48.3)	4 (33.3)	
Dyspnea					0.811
Yes	41 (53.9)	18 (51.4)	17 (58.6)	6 (50.0)	
No	35 (46.1)	17 (48.6)	12 (41.4)	6 (50.0)	
Effusion site					0.302
Left	26 (34.2)	16 (45.7)	6 (20.7)	4 (33.3)	
Right	43 (56.6)	16 (45.7)	20 (69.0)	7 (58.3)	
Bilateral	7 (9.2)	3 (8.6)	3 (10.3)	1 (8.3)	
Effusion volume					0.715
Small	27 (35.5)	13 (37.12)	10 (34.5)	4 (33.3)	
Moderate	19 (25.0)	11 (31.4)	6 (20.7)	2 (16.7)	
Large	30 (39.5)	11 (31.4)	13 (44.8)	6 (50.0)	
With other effusions					0.116
Pericardial effusion	14 (18.4)	4 (11.4)	9 (31.0)	1 (8.3)	
No	62 (81.6)	31 (88.6)	20 (69.0)	11 (91.7)	
Other metastasis					0.997
Lung	46 (60.5)	22 (62.9)	19 (65.5)	5 (41.7)	
Bone	36 (47.4)	14 (40.0)	16 (55.2)	6 (50.0)	
Liver	7 (9.2)	3 (8.6)	3 (10.3)	1 (8.3)	
Distant lymph nodes	9 (11.8)	4 (11.4)	4 (13.8)	1 (8.3)	
Brain	11 (14.5)	5 (14.3)	5 (17.2)	1 (8.3)	
TNM stage					0.642
IVa	34 (44.7)	17 (48.6)	11 (37.9)	6 (50.0)	
IVb	42 (55.3)	18 (51.4)	18 (62.1)	6 (50.0)	
NGS					0.556
Performed	19 (25.0)	7 (20.0)	8 (27.6)	4 (33.3)	
No or unknown	57 (75.0)	28 (80.0)	21 (72.4)	8 (66.7)	
Accompanied mutation					0.227
Present	14 (18.4)	4 (11.4)	6 (20.7)	4 (33.3)	
No or unknown	62 (81.6)	31 (88.6)	23 (79.3)	8 (66.7)	
NGS: next generation sequencing; EGFR: epidermal growth factor receptor; TNM: tumor-node-metastasis.					

其中19例患者进行了二代测序（next generation sequencing, NGS）检测，有14例患者存在共突变：7例仅合并*TP53*突变，占共突变者中的50%，1例合并CDK4/6扩增，1例合并*PIK3CA*突变，1例合并*CTNNB1*突变，1例合并*PIK3CA*、*TP53*、*FGFR3*、*SMAD4*突变，1例合并*CTNNB1*、*FGFR3*、*SMAD4*突变，1例合并*TP53*、*MET*突变，1例合并*TP53*、*RB1*、*MLH3*突变。

### 2.2 76例初诊*EGFR*阳性合并胸腔积液肺腺癌患者的治疗情况及疗效分析

76例患者中19例（25.0%）接受了化疗，19del、21L858R和非经典突变患者的化疗DCR分别为66.7%、50.0%和71.4%。57例（75.0%）患者接受了EGFR-TKIs治疗：49例（86.0%）接受一代药物治疗（包括吉非替尼、埃克替尼、厄洛替尼），19del、21L858R、非经典突变患者接受一代治疗的DCR分别为100.0%、82.6%、0%；6例（10.5%）接受了二代药物阿法替尼治疗，无21L858R患者，19del、非经典突变患者接受阿法替尼的DCR分别为100.0%和33.3%；2例（3.5%）接受了三代药物奥希替尼治疗，均为19del患者，DCR为100.0%。43例（56.6%）患者在一线治疗前或治疗期间接受了胸腔积液的局部处理，局部处理的患者中：仅行胸腔引流23例，占53.5%；胸腔注药14例，占32.5%，胸膜固定术6例，占14.0%。14例患者胸腔注射药物分别为：3例顺铂，2例顺铂+重组人血管内皮抑制素，2例重组人血管内皮抑制素，4例贝伐珠单抗，1例顺铂+贝伐珠单抗，1例顺铂+白介素-2，1例肿瘤坏死因子。具体治疗方法及疗效见表 2。

**表 2:** 76例初诊*EGFR*突变阳性合并胸腔积液肺腺癌患者的一线治疗方案及疗效[*n* (%)] First-line treatment and efficacy analysis of 76 lung adenocarcinoma patients harboring *EGFR* mutations with pleural effusion at initial diagnosis [*n* (%)]

Item	Total (*n*=76)	*EGFR*			*P*
		19del (*n*=35)	21L858R (*n*=29)	Non-classical mutations (*n*=12)	
Local treatment of pleural effusion					0.104
None	33 (43.4)	15 (42.9)	13 (44.8)	5 (41.7)	
Thoracic drainage	23 (30.3)	11 (31.4)	9 (31.0)	3 (25.0)	
Intrathoracic injection	14 (18.4)	3 (8.6)	7 (24.1)	4 (33.3)	
Chemical pleurodesis	6 (7.9)	6 (17.1)	0 (0.0)	0 (0.0)	
Effusion response					0.084
Increase	12 (15.8)	2 (5.7)	6 (20.7)	4 (33.3)	
Stable	12 (15.8)	7 (20.0)	2 (6.9)	3 (25.0)	
Reduce	30 (39.5)	14 (40.0)	14 (48.3)	2 (16.7)	
Disappear	22 (28.9)	12 (34.3)	7 (24.1)	3 (25.0)	
Control rate of effusion	84.2%	94.3%	79.3%	66.7%	0.040
First-line systemic treatment					0.001
1^st^-G TKI	49 (64.5)	24 (68.6)	23 (79.3)	2 (16.7)	
2^nd^-G TKI	6 (7.9)	3 (8.6)	0 (0.0)	3 (25.0)	
3^rd^-G TKI	2 (2.6)	2 (5.7)	0 (0.0)	0 (0.0)	
Chemotherapy	19 (25.0)	6 (17.1)	6 (20.7)	7 (58.3)	
First-line systemic response					0.008
PR	31 (40.8)	15 (42.9)	14 (48.3)	2 (16.7)	
SD	30 (39.5)	18 (51.4)	8 (27.6)	4 (33.3)	
PD	15 (19.7)	2 (5.7)	7 (24.1)	6 (50.0)	
DCR	80.3%	94.3%	75.9%	50.0%	0.003
mPFS (d)	273 (240.4-305.6)	287 (220.6-353.4)	327 (212.7-441.3)	55 (0-151.8)	0.001
TKI: tyrosine kinase inhibitor; CR: complete response; PR: partial response; SD: stable disease; PD: progressive disease; DCR: disease control rate; mPFS: median progression-free survival; 1^st^-G: first generation; 2^nd^-G: second generation; 3^rd^-G: third generation.					

### 2.3 12例*EGFR*非经典突变合并胸腔积液患者的临床特点

12例非经典突变患者中，外显子20插入突变（20ins）5例，20Q787Q突变3例，21非L858R突变3例。9例外显子20突变的患者中，3例（33.3%）选择靶向治疗，DCR为0%；6例（66.7%）选择化疗，DCR为83.3%。见表 3。

**表 3:** 12例*EGFR*非经典突变合并胸腔积液患者的临床特点 Clinical characteristics of 12 patients harboring *EGFR* non-classical mutations with pleural effusion at initial diagnosis

Case	Gender	Age (yr)	Mutation	Effusion amount	Effusion treatment	Effusion response	First-line treatment	Response	PFS (d)
1	Female	64	18G719X, 21L861Q	Large	Thoracic drainage	Increase	Gefitinib	PD	41
2	Male	70	21L861Q, TP53	Large	Thoracic drainage	Disappear	Afatinib	SD	549
3	Male	74	21A871V	Moderate	None	Increase	AC	PD	21
4	Male	65	20Q787Q	Small	None	Disappear	AC+Bev	SD	120
5	Female	64	20Q787Q	Large	Thoracic drainage	Increase	DP	PD	31
6	Male	60	20Q787Q	Large	DDP+Bev	Reduce	Afatinib	PD	55
7	Female	44	20V774M, 20H773L, *TP53*	Small	None	Disappear	AP	PR	190
8	Female	54	20ins, *PIK3CA*	Small	None	Increase	Gefitinib	PD	23
9	Male	39	20ins	Small	None	Stable	Afatinib	PD	31
10	Male	72	20ins	Moderate	Bev	Stable	AP	SD	98
11	Male	54	20ins	Large	DDP+Endostar	Stable	AP	SD	197
12	Male	66	20ins	Large	Bev	Reduce	AC+Bev	PR	256
AC: Pemetrexed+Carboplatin; AP: Pemetrexed+Cisplatin; DP: Docetaxel+Cisplatin; Bev: Bevacizumab.									

### 2.4 76例初诊*EGFR*阳性合并胸腔积液肺腺癌患者的生存分析

76例患者中，64例含有*EGFR*经典突变：接受靶向治疗的*EGFR* 19del和21L858R的患者mPFS分别为325 d *vs* 361 d（*P*=0.465）；接受化疗的*EGFR* 19del和21L858R的患者mPFS分别为92 d和70 d（*P*=0.901）。在12例非经典突变患者中，9例外显子20突变患者的mPFS为98 d：一线化疗和靶向治疗的mPFS分别为120 d和31 d（*P*=0.015）；一线化疗选择培美曲塞为主方案和多西紫杉醇为主方案的mPFS分别为190 d和31 d（*P*=0.025）。19例接受NGS检测的患者中，*EGFR* 19del、21L858R及非经典突变联合*EGFR*以外的共突变患者共有14例，mPFS分别为258 d、185 d和23 d，但无统计学差异（*P*=0.996）。全部76例患者的单因素生存分析显示PFS与患者的*EGFR*突变类型、胸腔积液控制情况、一线治疗药物、一线治疗疗效显著相关（*P* < 0.05），但与患者的性别、年龄、发病时有无呼吸困难、是否合并其他远处转移、胸腔积液部位、胸腔积液量、是否合并其他部位的积液、TNM分期、有无合并其他基因突变和胸腔积液的治疗方法均无显著相关（*P* > 0.05），见表 4和图 1。*Cox*多因素分析显示仅*EGFR*突变类型、一线治疗疗效是PFS的独立预后因素，见表 5。

**表 4:** 76例初诊*EGFR*突变合并胸腔积液肺腺癌患者的一线PFS单因素分析 Univariate analysis of PFS of first-line therapy in 76 lung adenocarcinoma patients harboring *EGFR* mutations with pleural effusion at initial diagnosis

Factor	*n*	mPFS (d)	*χ* ^2^	*P*	95%CI
Gender			2.409	0.121	
Male	32	211			117.4-304.6
Female	44	327			252.9-401.1
Age (yr)			0.038	0.846	
≤65	39	270			172.1-367.9
> 65	37	287			207.2-366.8
Dyspnea			2.872	0.090	
Yes	41	270			237.5-302.5
No	35	300			210.8-389.2
Lung metastasis			0.653	0.419	
Yes	46	285			210.6-359.4
No	30	256			229.2-282.8
Bone metastasis			0.107	0.744	
Yes	36	273			164.2-381.8
No	40	270			230.2-309.8
Liver metastasis			0.208	0.648	
Yes	7	98			26.1-169.9
No	69	285			243.0-327.0
Distant lymph node metastasis			0.114	0.735	
Yes	9	287			281.2-292.8
No	67	270			235.1-304.9
Brain metastasis			0.000	0.987	
Yes	11	285			155.5-414.5
No	65	270			233.5-306.5
Effusion site			1.523	0.467	
Left	26	287			218.3-355.7
Right	43	262			162.7-361.3
Bilateral	7	177			156.5-197.5
Effusion volume			1.120	0.571	
Small	27	273			176.3-369.7
Moderate	19	270			123.5-416.5
Large	30	285			182.6-387.4
With other effusion			0.772	0.380	
Pericardial effusion	14	417			182.6-651.4
No	62	262			206.3-317.7
TNM stage			0.080	0.778	
IVa	34	262			165.2-358.8
IVb	42	277			196.6-357.4
EGFR mutation type			13.722	0.001	
19del	35	287			220.6-353.4
21L858R	29	327			212.7-441.3
Non-classical mutations	12	55			0.000-151.8
Accompanied mutation			1.136	0.287	
Present	14	254			142.8-365.2
No or unknown	62	277			234.6-319.4
Effusion treatment			5.039	0.169	
None	33	273			172.8-373.2
Thoracic drainage	23	361			299.7-422.3
Intrathoracic injection	14	179			37.8-320.2
Chemical pleurodesis	6	287			240.2-333.8
Effusion response			103.664	< 0.001	
Increase	12	31			5.5-56.5
Stable	12	197			61.2-332.8
Reduce	30	277			239.3-314.7
Disappear	22	335			292.0-378.0
First-line treatment			25.681	< 0.001	
EGFR-TKIs	57	331			263.4-398.6
Chemotherapy	19	98			68.1-127.9
First-line response			118.566	< 0.001	
PR	31	361			309.0-413.0
SD	30	273			230.1-315.9
PD	15	41			24.0-58.0

**图 1:**
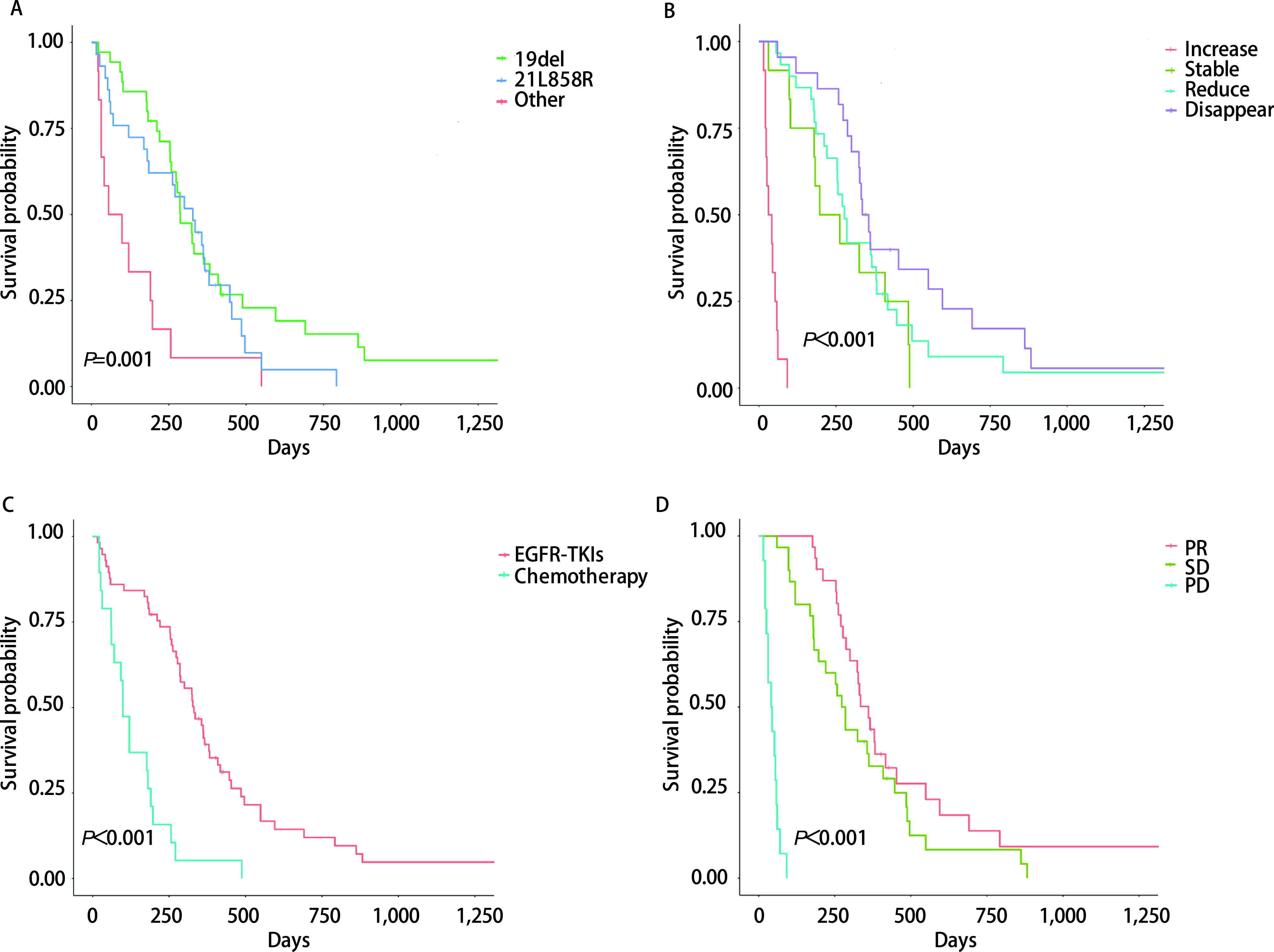
76例初诊*EGFR*阳性合并胸腔积液肺腺癌患者的PFS曲线。A：*EGFR* 19del、21L858R及其他*EGFR*突变亚型一线治疗的PFS；B：一线治疗期间胸腔积液不同控制程度组间的PFS；C：一线靶向治疗或化疗的PFS；D：一线治疗不同疗效组的PFS。 PFS curve of 76 lung adenocarcinoma patients harboring *EGFR* mutations with pleural effusion at initial diagnosis. A: The PFS of first-line therapy for *EGFR* 19del, 21L858R and non-classical mutations subtype; B: The PFS among patients with different response of pleural effusion during first-line therapy; C: The PFS of first-line targeted therapy or chemotherapy; D: The PFS of first-line therapy with different efficacy groups.

**表 5:** 76例*EGFR*突变初诊合并胸腔积液肺腺癌患者的一线PFS多因素分析 Multivariate analysis of PFS of first-line therapy in 76 lung adenocarcinoma patients harboring *EGFR* mutations with pleural effusion at initial diagnosis

Factor	*P*	HR	95%Cl
*EGFR* mutation type	0.034		
*EGFR* non-classical mutations	-	Reference	
*EGFR* 19del *vs* non-classical mutations	0.010	0.364	0.168-0.789
*EGFR* 21L858R *vs* non-classical mutations	0.031	0.419	0.190-0.924
Effusion response	0.335		
Effusion increase	-	Reference	
Effusion stable *vs* increase	0.369	0.508	0.116-2.228
Effusion reduce *vs* increase	0.342	0.527	0.140-1.978
Effusion disappear *vs* increase	0.125	0.317	0.073-1.377
First-line treatment			
Chemotherapy *vs* targeted therapy	0.060	1.960	0.973-3.947
First-line response	0.001		
PR	-	Reference	
SD *vs* PR	0.280	1.408	0.757-2.622
PD *vs* PR	< 0.001	92.894	8.832-977.055

## 3 讨论

*EGFR*基因是肺腺癌中最常见的驱动基因，其中外显子19缺失突变（19del）和外显子21点突变（21L858R）最常见，分别占*EGFR*所有突变的45%和40%，被称为经典突变^[7]^，非经典突变中最常见的3种是外显子20插入突变（20ins），外显子18 G719X突变（18G719X），外显子21 L861Q突变（21L861Q），分别占所有*EGFR*突变的4.9%-12%、3%-4%和1%^[8]^。复合突变是指同时检测到两种或两种以上*EGFR*突变，占所有*EGFR*突变的2.75%-14%^[9]^。共突变是指同时检测到*EGFR*和其他基因突变，最常见的为合并*TP53*突变，发生率为25%-44%^[10-12]^。本研究中经典突变19del和21L858R突变分别占46.0%和38.2%；非经典突变占15.8%，其中前三位分别是20ins（6.6%）、20Q787Q（3.9%）、21L861Q（2.6%）；*EGFR*复合突变患者2例（2.6%）；虽然NGS检测技术更有利于发现*EGFR*复合突变和共同变^[13]^，但因NGS检测价格较为昂贵、进入临床时间较短，本研究19例NGS检测患者中14例含有共突变，涉及*TP53*、*CDK4/6*、*PIK3CA*等，其中仅合并*TP53*突变的占50.0%。本研究各类型突变的发生率与既往文献^[8, 10-12]^基本一致，提示合并胸腔积液的*EGFR*突变阳性肺腺癌在*EGFR*的表达分布上并无特殊；本研究还进一步探讨了初诊时*EGFR*非经典突变合并胸腔积液的肺腺癌患者的具体突变类型，但样本量较小。关于初诊*EGFR*突变阳性合并胸腔积液患者各突变亚型的发生率、共突变情况仍需要大样本研究验证。

本研究中各*EGFR*突变类型间的临床特征无显著差异，恶性胸腔积液的局部治疗方式的比例无明显差异，均有40%以上的患者未进行任何胸腔治疗。不同突变类型的胸腔积液控制率存在显著差异，19del、21L858R和非经典突变患者的胸腔积液控制率分别为94.3%、79.3%、66.7%（*P*=0.040），与全身治疗的DCR呈一致趋势。生存分析显示，初诊时胸腔积液量和胸腔积液局部处理方式、胸腔积液控制效果均与一线治疗PFS无关。既往的一项观察性队列研究^[14]^显示，在接受EGFR-TKIs治疗的*EGFR*突变阳性的NSCLC患者中，早期滑石粉胸膜固定术可能不会降低胸腔积液复发率。一项中国研究^[15]^显示，接受埃克替尼联合胸膜固定术的患者中位PFS为8.4个月，而单独接受埃克替尼治疗的患者中位PFS为9.0个月（*P*=0.996）；两组客观缓解率（objective response rate, ORR）也没有显著差异（64.29% *vs* 67.57%, *P*=0.824）。日本的一项研究^[16]^则认为*EGFR*突变阳性肺腺癌合并恶性胸腔积液的患者仅接受一线EGFR-TKIs治疗可能比TKI治疗同时接受胸膜固定术的患者预后更好，总生存期（overall survival, OS）为31.1个月*vs* 21.8个月。结合既往文献和本研究结果，提示相比局部治疗，全身治疗效果是影响患者胸腔积液控制率的重要因素，优化一线治疗方案显得尤为重要。

在*EGFR*基因敏感突变的晚期肺腺癌患者中，一线使用EGFR-TKIs治疗已列入多个指南，本研究因含有部分非经典突变患者，且入组时间跨度大，在靶向治疗药物进入中国医保之前，部分患者一线使用了化疗。单因素分析示一线靶向治疗和化疗的PFS分别为331 d *vs* 98 d（*P* < 0.001）；但多因素分析仅*EGFR*突变类型及一线疗效与一线PFS有关，19del和21L858R对比非经典突变，其疾病进展风险明显下降，HR分别为0.364和0.419。19del和21L858R两组患者，无论接受靶向治疗还是化疗，PFS均无统计学差异。一项荟萃分析^[17]^显示，对于接受TKI治疗的*EGFR*突变阳性非小细胞肺癌患者，19del突变患者的PFS优于21L858R突变患者（HR=0.59, 95%CI: 0.38-0.92, *P*=0.019）。但在另一项临床研究中，中国学者Zheng等^[18]^回顾性分析了203例在初始或一线进展后出现恶性胸腔积液的*EGFR*突变阳性的非小细胞肺癌患者，19del突变和21L858R突变患者一线使用TKI治疗后PFS分别为9.4个月和8.8个月（*P*=0.53）。可能原因是：合并胸腔积液总体预后差，导致PFS差异不明显^[19]^；合并恶性胸腔积液者EGFR-TKIs治疗敏感性下降^[20]^。对于非经典突变，不同类型的突变适合的治疗不同，如18G719X、20S768I、21L861Q对于二代EGFR-TKI阿法替尼更敏感，20ins对EGFR-TKIs敏感性均差，更适合首选化疗^[8]^。本研究非经典突变患者中以外显子20突变居多，主要为20ins和20Q787Q突变，患者一线治疗方案以化疗为主，占66.7%；9例外显子20突变患者中，靶向治疗的DCR为0，化疗的DCR为83.3%，靶向治疗和化疗的PFS分别为31 d和120 d（*P*=0.015）；在化疗方案的选择上，培美曲塞为主的方案对比多西紫杉醇方案，PFS分别为190 d和31 d（*P*=0.025）。既往文献^[10-12, 21]^显示存在共突变的患者生存期和药物敏感性大多较差，但本研究中19del、21L858R及非经典突变发生共突变率无统计学差异，患者PFS无统计学差异，可能与本研究样本量较少有关。总体而言，对于*EGFR*经典突变及非经典突变合并胸腔积液肺腺癌患者的治疗方案仍有待进一步探讨和优化。

本研究不足之处有：①本研究为单中心的回顾性研究，样本量偏小，可能存在选择偏倚；②OS由于随访时间不够长，失访、删失比例高，未对OS进行比较；③胸腔积液量的分类目前尚无基于CT图像的定量标准，由本研究的研究者参考既往文献^[22-24]^设定，因肺癌患者确诊后往往不再做X片，已有的文献^[25]^也显示胸片在检测积液方面不太敏感，故未按教科书上胸片定量的标准来界定胸腔积液量，未来可考虑应用体积计算法进行精准分型。

综上，初诊时*EGFR*突变阳性且合并胸腔积液的肺腺癌患者，*EGFR*不同突变亚型的临床特征无显著区别，胸腔局部治疗方式在不同突变亚型及一线PFS中未见显著差异，经典突变19del和21L858R的胸腔积液控制率显著优于非经典突变患者，与全身DCR一致；*EGFR*突变亚型和一线治疗疗效是一线PFS的独立预后因素。因此，未来在精准检测的前提下，优化一线治疗方案，提高一线疗效，是延长*EGFR*突变阳性合并胸腔积液肺腺癌患者PFS的关键。
